# A Case of Incomplete and Atypical Kawasaki Disease Presenting with Retropharyngeal Involvement

**DOI:** 10.3390/ijerph16183262

**Published:** 2019-09-05

**Authors:** Chiara Isidori, Lisa Sebastiani, Susanna Esposito

**Affiliations:** 1 Department of Surgical and Biomedical Sciences, University of Perugia, 06132 Perugia, Italy (C.I.) (L.S.); 2 Department of Medical and Surgical Sciences, University of Parma, 43126 Parma, Italy

**Keywords:** abscess-like lesions, Kawasaki disease, lymphadenopathy, neck swelling, retropharyngeal involvement

## Abstract

**Background:** Kawasaki disease (KD) is a childhood acute febrile vasculitis of unknown aetiology. The diagnosis is based on clinical criteria, including unilateral cervical lymphadenopathy, which is the only presenting symptom associated with fever in 12% of cases. A prompt differential diagnosis distinguishing KD from infective lymphadenitis is therefore necessary to avoid incorrect and delayed diagnosis and the risk of cardiovascular sequelae. **Case presentation:** We describe the case of a 4 years old boy presenting with febrile right cervical lymphadenopathy, in which the unresponsiveness to broad-spectrum antibiotics, the following onset of other characteristic clinical features and the evidence on the magnetic resonance imaging (MRI) of retropharyngeal inflammation led to the diagnosis of incomplete and atypical KD. On day 8 of hospitalisation (i.e., 13 days after the onset of symptoms), one dose of intravenous immunoglobulins (IVIG; 2 g/kg) was administered with rapid defervescence, and acetylsalicylic acid (4 mg/kg/day) was started and continued at home for a total of 8 weeks. Laboratory examinations revealed a reduction in the white blood cell count and the levels of inflammatory markers, thrombocytosis, and persistently negative echocardiography. Clinically, we observed a gradual reduction of the right-side neck swelling. Fifteen days after discharge, the MRI of the neck showed a regression of the laterocervical lymphadenopathy and a resolution of the infiltration of the parapharyngeal and retropharyngeal spaces. **Conclusion:** Head and neck manifestations can be early presentations of KD, which is frequently misdiagnosed as suppurative lymphadenitis or retropharyngeal infection. A growing awareness of the several possible presentations of KD is therefore necessary. Computed tomography (CT) or MRI can be utilised to facilitate the diagnosis.

## 1. Introduction

Kawasaki disease (KD) is an acute self-limiting vasculitis that occurs in childhood, also known as acute febrile mucocutaneous lymph node syndrome, described for the first time by Dr. Tomikasu Kawasaki in 1967. The disease involves small and medium-sized blood vessels and leads to coronary artery aneurysms in approximately 25% of untreated cases, with increased risks of cardiovascular complications and mortality [1]. KD has an overall incidence in the United States of 18–21 per 100,000 and in Europe of 3–10 per 100,000, with a higher incidence in Japan estimated at 76–90 per 100,000 [2,3]. KD typically affects children under 5 years of age with a peak age incidence between 6 months and 2 years [4,5,6,7].

The pathogenesis of KD is related to an abnormal immune response to environmental and infectious triggers [8,9,10]. The diagnosis is based on clinical criteria, including fever for at least 5 days and the presence of four out of the following five clinical signs: non-exudative bulbar conjunctivitis, erythema of the lips and oral mucosa, changes in peripheral extremities, polymorphous rash and cervical lymphadenopathy > 1.5 cm [5]. An early diagnosis, which is useful for reducing the risk of coronary artery abnormalities, is often difficult because the disease frequently presents in an incomplete (i.e., fewer than four clinical criteria) or atypical form (i.e., signs and symptoms different from the characteristic clinical features) with an absence of pathognomonic symptoms and specific diagnostic laboratory tests [6].

Lymphadenopathy occurs in 50–70% of patients with KD, which is less frequent than the prevalence of the other diagnostic criteria that are present in approximately 90% of cases [4]. Cervical lymphadenopathy with fever appears as an initial symptom in approximately 12% of cases. Therefore, a differential diagnosis distinguishing between KD and bacterial lymphadenitis is critical, as the therapies and outcomes differ considerably [11,12]. Moreover, neck lymphadenopathy is rarely associated with deep neck inflammation, appearing with an atypical retropharyngeal abscess-like lesion that can lead to a delayed diagnosis [13]. In this report, we describe a young boy with para-retropharyngeal tissue inflammation that was not responsive to antibiotic therapy, but promptly responded to immunoglobulin treatment. Incomplete and atypical KD was diagnosed.

## 2. Case Report

A 4 year old boy presented to our paediatric unit with a 5 day history of right laterocervical swelling and neck pain without restriction of movement, dysphagia or dyspnoea, and a 3 day history of fever. Antibiotic therapy with amoxicillin-clavulanate was started and continued for 72 h without clinical improvement. Physical examination showed right-side, painful, tender, tense–elastic laterocervical lymphadenopathy with no inflammation of the overlying skin and non-suppurative pharyngotonsillitis. The remaining superficial lymph nodes were not affected and the liver and spleen were palpable at the costal margin. The initial laboratory tests showed neutrophilic leucocytosis, an increase in the levels of inflammatory markers (i.e., white blood cell count, 16,750/µL; neutrophils, 72.4%; C reactive protein, 4 mg/dL; erythrocyte sedimentation rate, 48 mm/1 h) and ferritin in the normal range (i.e., 73 ng/mL). The neck ultrasound highlighted multiple hypoechoic lymph nodes in the right anterolateral compartment, with a tendency towards confluence and a maximum diameter of 34 mm that displaced muscular and vascular bundles. 

On the first day of hospitalisation, intravenous antibiotic therapy was started with ceftriaxone, vancomycin and clarithromycin based on the suspicion of suppurative lymphadenitis. The blood peripheral smear, blood culture and infectious serology, including Epstein-Barr, cytomegalovirus, *Toxoplasma gondii, Bartonella henselae*, *Mycoplasma pneumoniae* and Adenovirus, were negative. Abdominal ultrasounds and chest radiography were normal. 

**Figure 1:**
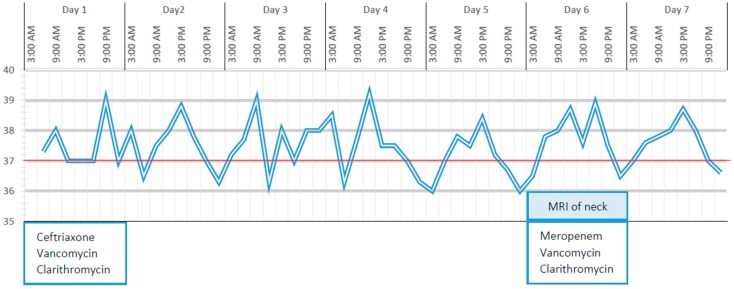
Body temperature and antibiotic therapy during the first 7 days of hospitalisation.

**Figure 2:**
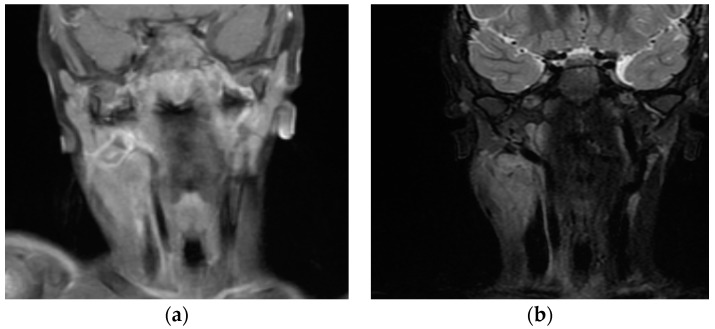
Magnetic resonance imaging (MRI) of the neck. (**a**): Right anterolateral multiple polycyclic lymph nodes with ill-defined contours and tendency to confluence. Intense enhancement of the adjacent sternocleidomastoid. Displacement of the vascular bundle of the neck and internal jugular vein compression. (**b**): Enhancement of parapharyngeal and retropharyngeal tissues without fluid collection and with preserved respiratory space. Multiple bilateral enlarged lymph nodes in parapharyngeal and retropharyngeal space. Minimum contrast enhancement of the atlantoaxial joint.

On day 6 of hospitalisation, due to the persistence of daily fever, the elevation of the level of C reactive protein (8 mg/dL) and the lack of clinical or ultrasound improvements in the neck lymphadenopathy, antibiotic therapy was implemented with the replacement of ceftriaxone with meropenem (Figure 1). Magnetic resonance imaging (MRI) of the neck was performed and confirmed the presence of multiple right-side lymph nodes with a tendency towards confluence and an infiltration with intense enhancement of the sternocleidomastoid, parapharyngeal and retropharyngeal tissues with preserved respiratory space, which was initially interpreted as an infectious complication (Figure 2). In the previous 2 days, the child had developed non-exudative conjunctivitis, an intermittent micro-macular rash of the trunk, arthralgias in multiple joints and arthritis of the right knee, as documented by ultrasound. 

Based on this clinical presentation (i.e., more than 5 days of fever associated with cervical lymphadenopathy, conjunctivitis, polymorphous rash, arthralgias/arthritis and the presence of retropharyngeal tissue inflammation) and on the lack of response to different broad-spectrum antibiotics, incomplete KD was hypothesised. The echocardiography performed on the sixth day did not show coronary artery abnormalities. No evidence of uveitis was found on the eye exam. 

**Figure 3:**
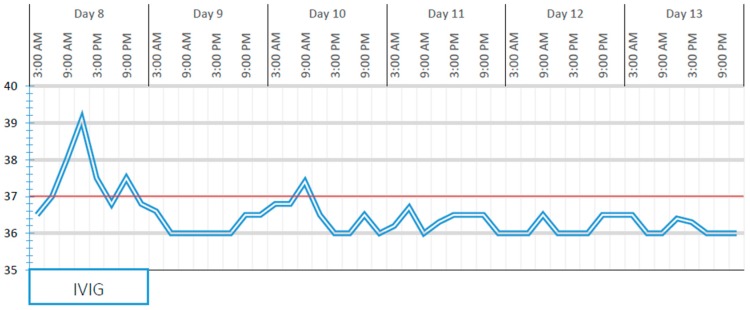
Body temperature from the time of the administration of intravenous immunoglobulin (IVIG) treatment.

On day 8 of hospitalisation (i.e., 13 days after the onset of symptoms), one dose of intravenous immunoglobulins (IVIG; 2 g/kg) was administered with rapid defervescence (Figure 3), meropenem was stopped once the fever disappeared (i.e., it was administered for 4 days) and acetylsalicylic acid (4 mg/kg/day) was started and continued at home for a total of 8 weeks. Laboratory examinations on day 12 revealed a reduction in the white blood cell count and the levels of inflammatory markers (white blood cell count, 5180/µL; C reactive protein, < 0.4 mg/dL), thrombocytosis (platelets, 548,000/µL), and persistently negative echocardiography. Clinically, we observed a gradual reduction of the right-side neck swelling. Therefore, in our case, deep neck inflammation was associated with KD, which was diagnosed based on the response to immunoglobulin treatment.

Fifteen days after discharge, the MRI of the neck showed a regression of the laterocervical lymphadenopathy and a resolution of the infiltration of the parapharyngeal and retropharyngeal spaces.

The management of this patient was approved by the Ethics Committee of Umbria Region (PED-2019-08), and both parents provided written informed consent for the evaluation of the child. The Ethics Committee of Umbria Region approved the publication of this case, and both parents provided written informed consent for the publication of this manuscript, including MRI imagingphotos.

## 3. Discussion

The prompt diagnosis of KD remains a challenge for clinicians who have to be alert to identifying head and neck manifestations with a poor response to broad-spectrum intravenous antibiotic therapy as possible early presentations of KD. This case report shows that retropharyngeal involvement with oedema, cellulitis and abscess-like lesions is a rare but possible manifestation of KD. Computed tomography (CT) or MRI can be utilised to facilitate the diagnosis.

Cervical lymphadenopathy is a frequent pathological condition in children. A careful differential diagnosis, especially among infectious diseases, KD and lymphoproliferative disorders, is fundamental to avoid incorrect and delayed diagnosis [11,12]. In KD, cervical lymphadenopathy is usually unilateral and confined to the anterior cervical triangle. Imaging studies of the neck, comprising ultrasound, CT and MRI, can be useful in certain cases to distinguish between cervical lymphadenopathy in KD and bacterial infection. However, a previous study suggested that lymphadenopathy in KD is initially diagnosed as bacterial lymphadenitis and treated with antibiotics in approximately 80% of patients [14]. Similarly, in our case, the initial clinical presentation with fever and unilateral right lymphadenopathy led to the diagnosis of bacterial lymphadenitis, which was supported by laboratory exams and ultrasound images. 

Regarding ultrasound for cervical lymphadenopathy, recent studies suggest that there are no sensitive or specific findings in patients with KD to differentiate this disease from bacterial lymphadenitis, even if the more commonly detected characteristics are multiple hypoechoic enlarged nodes with normal echogenic hilum and well-defined contour forming a mass known as a “cluster of grapes” [15,16]. Purulent lymphadenopathy is frequently characterised by lymph nodes with the loss of normal hilum, poorly defined margins and a heterogeneous echotexture. After 6 days of hospitalisation (i.e., 11 days after the onset of symptoms), we performed MRI on the neck due to the suspicion of a deep neck suppurative complication. We found multiple anterolateral cervical lymph nodes with heterogeneous enhancement, consistent with a suppurative-like lesion and a para-retropharyngeal enhancement extending to the contiguous tissues without evidence of abscess that we initially interpreted as an extension of the bacterial infection. Although persistent unresponsiveness to antibiotics after 8 days of broad-spectrum therapy led us to consider a different diagnosis that took into account the onset of other characteristic clinical features, the rapid resolution of the fever after the administration of IVIG confirmed the correct diagnosis of KD.

The associations of cervical lymphadenopathy, retropharyngeal oedema, cellulitis or abscess-like lesions in KD have been increasingly described [1,17]. Deep neck inflammation is found in approximately 5% of head and neck manifestations in KD and is most likely underestimated due to the fact that signs and symptoms of retropharyngeal involvement, such as stridor, neck pain and dysphagia, are less common in KD than in retropharyngeal abscesses. Only a small proportion of patients undergo imaging studies [18,19].

The retropharyngeal abnormalities in KD are presumably linked to the vasculitis of microvessels that cause oedema and inflammation [20]. In the literature, CT is the imaging study of choice used to identify retropharyngeal abnormalities and to differentiate abscesses from KD inflammation in the majority of cases. The principal findings considered specific for a suppurative complication are ring enhancement, low-density core, soft tissue swelling and mass effects, demonstrating some limitations and a lack of specificity [19,21]. We performed MRI of the neck to aid in the differential diagnosis. MRI is very useful for the investigation of soft tissues and does not expose the patient to ionizing radiation, although it requires sedation in young or uncooperative children. On the MRI, suppurative lymphadenitis appears as central T1 hypointensity, T2 hyperintensity and peripheral enhancement [12]. To date, we found limited data in the literature regarding MRI in cervical lymphadenopathy and retropharyngeal involvement in KD. The principal findings reported in four cases were retropharyngeal and upper jugular lymphadenopathy with capsular and hilar enhancement, perinodal and muscular infiltration, a retropharyngeal oedema pattern with contrast enhancement, atlantoaxial effusion and synovial enhancement in the temporomandibular joint [22].

In our case, an initial erroneous diagnosis of cervical bacterial lymphadenopathy delayed the correct identification of incomplete and atypical KD when other characteristic features appeared sequentially. The MRI images showed the presence of retropharyngeal involvement without clinical response to antibiotic therapy, but a rapid response to IVIG treatment.

## 4. Conclusions

Our case showed that persistent febrile cervical lymphadenopathy with unresponsiveness to broad-spectrum antibiotics, and the evidence on the MRI of retropharyngeal inflammation should lead to consideration of incomplete and atypical KD. A growing awareness of the several possible presentations of KD is therefore necessary. CT or MRI can be utilised to facilitate the diagnosis. This report is very valuable for practitioners, because cervical lymphadenopathy and fever are usually common in bacterial infections, and antibiotics are prescribed as first-line treatment. If no effect is observed, KD must be considered if the fever lasts, other symptoms occur and inflammatory markers increase.
